# The Association of Short-Video Problematic Use, Learning Engagement, and Perceived Learning Ineffectiveness among Chinese Vocational Students

**DOI:** 10.3390/healthcare11020161

**Published:** 2023-01-05

**Authors:** Jian-Hong Ye, Yu-Feng Wu, Weiguaju Nong, Yu-Tai Wu, Jhen-Ni Ye, Yu Sun

**Affiliations:** 1 Faculty of Education, Beijing Normal University, Beijing 100875, China; 2 National Institute of Vocational Education, Beijing Normal University, Beijing 100875, China; 3 Office of Physical Education, Ming Chi University of Technology, New Taipei City 243303, Taiwan; 4 School of Education, Guangxi University of Foreign Languages, Nanning 530222, China; 5 Office of Physical Education, Soochow University, Taipei City 111002, Taiwan; 6 Graduate Institute of Technological & Vocational Education, National Taipei University of Technology, Taipei City 106344, Taiwan; 7 Department of Industrial Education, National Taiwan Normal University, Taipei City 106, Taiwan

**Keywords:** behavioral–emotional–cognitive, ecological systems theory, engagement theory, learning engagement, perceived learning ineffectiveness, person–process–content, short-video addiction, short-video problematic use

## Abstract

Short videos are very popular among students, but the immersive nature of the software makes them prone to problematic use and even addiction. Internet addiction, including short-video problematic use, has been a hot topic in recent years due to the COVID-19 epidemic. However, there are few empirical studies that have explored the effects of short-video problematic use on students. Thus, vocational colleges in China were recruited to participate in this study. There were 1089 effective participants, with a valid recall rate of 90.8%. This included 466 male students (42.8%) and 623 female students (57.2%), with a mean age of 19.19 years (standard deviation of 1.07 years). Five hypotheses were proposed and validated by structural equation modeling within the framework of ecological systems theory and engagement theory to explore the association of short-video problematic use, three types of learning engagement, and perceived learning ineffectiveness. Research findings showed that: (1) short-video problematic use has a negative effect on behavioral engagement; (2) behavioral engagement has a positive effect on both emotional and cognitive engagement; and (3) emotional and cognitive engagement have a negative effect on perceived learning ineffectiveness. According to the results, it can be seen that short-video problematic use has a detrimental effect on students’ learning experiences, so teachers and parents need to pay attention to the negative effects of excessive use among students.

## 1. Introduction

In recent years, short-video applications have become popular worldwide, and although this software encourages people to communicate and interact via the Internet [1], excessive use of these applications may lead to addiction. However, watching online videos (including short videos) has become the most popular leisure activity in China [2], and short-video applications such as TikTok and Kuaishou are becoming increasingly popular in China. The number of people addicted to short videos is also increasing [3]. Because social media is a platform where ideas, opinions, feelings and information can be easily exchanged, it leads to students spending most of their time using different media applications and eventually becoming addicted [4]. In addition, the popularity of this software has contributed to the over-addiction of adolescents, with short-video problematic use existing to some extent among adolescents [5]. This excessive use of short-video apps can be considered to be a state in which people spend a great deal of time using these apps, even though they are aware of the negative outcomes that may occur by doing so [1]. The use of short videos raises new concerns [6]. Therefore, it is an emerging Internet addiction, which refers to the inability of short-video users to use the software reasonably, to the extent that the desire to use it will affect their normal daily life.

Despite the growing impact of short videos, there is a lack of research on short-video problematic use [5]. Little is known about the psychological mechanisms associated with TikTok use [7]. This also makes short-video problematic use an emerging and under-researched public health problem. Because of the highly immersive nature of short videos, users are more likely to find it difficult to extricate themselves, so it is necessary to explore this issue in more depth. The Internet has become an important part of daily life for young students, as people of the Internet nation. Thus, this research explores how short-video problematic use affects students’ academic engagement and effective learning.

Bronfenbrenner proposed the ecological systems theory (EST) to explain the interaction between humans and the environment, arguing that an individual’s world can be theorized as an ecological system [8]. This theory has been used to investigate the interaction among person–process–content (PPC) [9]. The core analysis of mental health research that applies Bronfenbrenner’s theory examines how mental health is determined through the interactions between individual factors and the ecosystems surrounding the individual/group and the interactions between and within these ecosystems [10]. Furthermore, the EST framework uses the interaction between student characteristics and background as a driver of student experience and outcomes and thus can help explain the pathways through which students can achieve academic success [11]. Therefore, this study explored the impact of short-video problematic use on student learning through the lens of the EST-based PPC model.

It is now widely accepted that the emphasis on effective learning is based on the individual. Therefore, effective learning requires students to be actively engaged in learning activities through which they internalize what they have learned and are able to absorb and reflect on their learning experiences [12]. Generally, students engage in a learning activity by interacting, acting, trying, thinking, and talking with their classmates and teachers [13]. Thus, engagement is assumed to be malleable, responsive to environmental features and adaptable to environmental changes. Thus, it is also thought to encompass three dimensions, namely behavioral, emotional and cognitive, and is proposed for study as a multidimensional construct [14]. In educational contexts, behavioral engagement represents learner effort, persistence, engagement, and perseverance, which are critical to achieving positive academic outcomes and preventing dropping out [15]. On the other hand, emotional engagement is a form of participation that addresses the level of motivation, satisfaction, comfort and interest that young people have in their classes and their desire to achieve academic success [16]. On the other hand, cognitive engagement refers to a student’s level of commitment to learning, which involves being thoughtful and determined in approaching tasks from school and being ready to put in the essential effort required to know complicated ideas or master challenging skills [14]. Therefore, learning engagement is considered critical to effective learning because it has a positive impact on learning outcomes [12]. However, the process from learner engagement to learning outcomes still requires more specific research [15]. Based on the above, this study considers multidimensional learning engagement to be an important factor influencing learning, and therefore, this dimension is one of the variables.

The aim of the activity determines the learning outcomes, especially whether the desired results are achieved [17]. However, young students tend to evaluate their learning effectiveness from a negative perspective [18], which means learning ineffectiveness. However, in the past, most research has focused on student learning outcomes. The issue of ineffective learning has only begun to receive attention since the onset of COVID-19 [19], so exploration of the factors that contribute to ineffective learning is still rare. In addition, studies on the topic of short-video problematic use have not been conclusive about the effect of addiction on learning ineffectiveness. However, understanding how to promote and inhibit ineffective learning is critical to improving educational outcomes. Therefore, this study used perceived learning ineffectiveness as a way for vocational students to express their perceptions of whether the learning outcomes met their needs after using the short videos.

In summary, within the framework of EST and engagement theory, the purpose of this study was to examine the relationship between short-video problematic use as a “person,” the three types of learning engagement as a “process,” and perceived learning ineffectiveness as “content” in order to construct a research model based on a cross-sectional design scope. Five research hypotheses were proposed to examine the associations between short-video problematic use, the three types of learning engagement, and perceived learning ineffectiveness.

## 2. Research Model and Hypothesis

### 2.1. Theoretical Background

EST adopts a life course method to know how improvement happens throughout increasingly complex procedures of contact between people and their environment [20]. It helps explain how individuals and the environment they live in are synergistic and interdependent, so this theory is largely regarded as an environmental theory [21]. In the framework of EST, ecology indicates the fit between individuals and the environment, which not only promotes the improvement of the environment, but additionally gives people an environment to grow in. It is, therefore, thought that there is a close association between people and the environment. Thus, EST assumes that individual development is a dynamic process involving interactions between individuals and the systems in which they operate [22]. Moreover, Bronfenbrenner encourages educationalists and psychologists to see people as central to such complex systems and to recognize and analyze the systems that make up their environment and how they interact in trying to understand people and their personal challenges [23]. EST is now one of the key frameworks in educational research, helping to explain how people produce corresponding educational outcomes as a result of the influence of environmental systems. Therefore, EST can also help explain how students’ addiction to short videos can affect their learning environment and further contribute to the outcome.

### 2.2. Research Model

**Figure 1:**
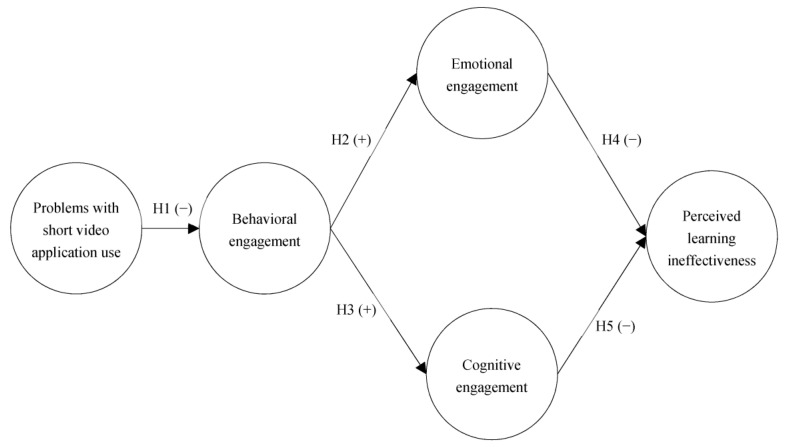
Research model.

The PPC model of EST can help explain the impact of addiction in the educational setting [24]. Therefore, under the framework of EST and engagement theory, for this study, we proposed five hypotheses and constructed a model to explore the association among short-video problematic use, three types of learning engagement, and perceived learning ineffectiveness, which can be seen in Figure 1.

### 2.3. Research Hypothesis

Many studies have been conducted in the past to confirm the negative effects of problematic cell phone use, problematic Internet use, and excessive game playing on the student population. The problematic use of short-video applications has also gained attention in the past few years, and studies have gradually begun to confirm its adverse effects on students’ academic life. Therefore, this study proposed the following five hypotheses based on the theory and the related literature.

#### 2.3.1. Short-Video Problematic Use Is Negatively Related to Behavioral Engagement

The addiction process can be defined as a transition between patterns of behavioral control [25]. Many people are addicted to watching short videos on their cell phones and thus neglect their normal school life and work due to their preoccupation with the Internet. This, in turn, negatively affects their academic performance [3]. In addition, it has been suggested that smartphone addiction can negatively affect students’ learning [26] because students’ attention in class is disturbed by the use of their phones, and this distraction also affects their learning outside of class [27]. In addition, it has been found that Internet addiction is a significant predictor of school engagement [28]. Some studies have also established that Internet addiction is negatively related to academic engagement [29]. Therefore, this study used short-video problematic use to investigate the association between participants’ behavioral engagement with the following hypothesis:

**H1.**  *Short-video problematic use is negatively related to behavioral engagement*.

#### 2.3.2. Behavioral Engagement Is Positively Related to Emotional and Cognitive Engagement

Behavioral, emotional, and cognitive engagement are considered to be content-specific and, by extension, lead to the identification of different determinants and outcomes [30]. In different contexts, they all emphasize the importance of different dimensions of engagement to the particular outcome sought [31]. In the educational context, engagement refers to the process of students’ effective participation in learning activities, and engagement in educational settings is conceptualized as three components: emotional, behavioral, and cognitive, which are conceptually separate but related [32]. Furthermore, previous research has indicated that a strong relationship between behavioral, emotional, and cognitive engagement is often observed [33]. This is because, without engagement, students emotionally and cognitively retreat from the learning process [34]. Furthermore, the study by Luan et al. confirms the role of behavioral engagement in influencing the remaining engagement dimensions [35].

When students feel bored or uninterested in a task, they will also be emotionally detached from the task. In addition, when they are not willing to listen to other members’ reactions or engage in interactions, they are less likely to exert effort or persistence or direct attentional resources in an effective manner for cognitive engagement [31]. Therefore, it is reasonable to assume that when students are not behaviorally engaged, it would be difficult for them to become emotionally and cognitively engaged. Taken these factors together, this study used behavioral engagement to examine the associations of participants’ emotional and cognitive engagement, with the following hypotheses.

**H2.**  *Behavioral engagement is positively related to emotional engagement*.

**H3.**  *Behavioral engagement is positively related to cognitive engagement*.

#### 2.3.3. Emotional and Cognitive Engagement Are Negatively Related to Perceived Learning Ineffectiveness

Student engagement is a hotly debated topic in all areas of higher education, and it is considered to have a clear and potential relationship with assessment outcomes [36]. This is because once students are well engaged, student learning ineffectiveness is somewhat alleviated [37]. In addition, student engagement is also considered to be related to the success of school, educational, and social activities, which is important for avoiding academic failure and improving competence, which influences a broad range of youths’ outcomes [33].

Whereas emotional engagement is conceptualized as a sense of investment and identification, identification is a sense of belonging, feeling that school is an essential factor of learning, and finding value in the school experience [34]. The evolutionary view of emotion suggests that valuable environmental events should be easily prioritized for perceptual processing, and this can be achieved by increasing the detection of emotional events by enhancing attention through emotion [38]. Therefore, when students are emotionally engaged in the learning process, it increases their sense of value and focus on the learning task, leading to better learning outcomes.

In addition, a cognitively engaged student will be one who is willing to engage in learning and accept the challenge of acquiring new knowledge or skills and exerting effort beyond the requirements of the course [36]. This is because students become mentally engaged when they make cognitive efforts to understand, exceed activity requirements, solve problems flexibly, and select challenging tasks [39]. Therefore, self-reflection can enhance their strengths and improve their learning outcomes [16]. Having good cognitive engagement will thus help students achieve good learning outcomes. Taking these factors together, this study examined the relationship between participants’ perceived learning ineffectiveness in terms of emotional and cognitive engagement, with the following hypotheses:

**H4.**  *Emotional engagement is negatively related to perceived learning ineffectiveness*.

**H5.**  *Cognitive engagement is negatively related to perceived learning ineffectiveness*.

## 3. Methods

### 3.1. Procedure

This study was conducted by creating an online questionnaire through the Wenjuanxing platform (one of the most well-known questionnaire platforms in China) and used the snowball sampling method to send the questionnaire link to exchange groups of university students in Chinese vocational colleges via messaging applications such as WeChat and QQ, inviting students who use short-video applications to complete the questionnaire and send it to their classmates. In addition, it was also stated that students from vocational institutions who were using short-video applications could participate in this study. If they did not meet these criteria, they could not complete the survey. In addition, no incentives were provided to participate. This study was based on a cross-sectional design, and questionnaires were collected on 1 June 2022. The questionnaire link was shut down after 1200 questionnaires were received.

In this study, all research processes, including questionnaire collection, were conducted according to American Psychological Association ethical principles and China’s regulatory ethical requirements. We provided a statement of the aim of the study, how data would be used, participant rights and privacy protection, and a contact email address of the author on the front page of the web-based questionnaire. By agreeing to complete the questionnaire, participants were deemed to have signed an electronic informed consent statement.

### 3.2. Participants

This study collected 1200 questionnaires, of which 111 were excluded due to being incomplete or taking less than 5 min to complete. This left a final sample of 1089, including 466 male students (42.8%) and 623 female students (57.2%); 743 (68.2%) were enrolled in a vocational school, and 346 (31.8%) were enrolled in a university of science and technology. The average number of days per week of short-video application use was 675 (62%) for daily users, 277 (25.4%) for 4~6 days, and 137 (12.6%) for 1–3 days. The average number of minutes of use was 97 (8.9%) for less than 1 h, 609 (55.9%) for 1–3 h, 334 (30.7%) for 3–5 h, and 49 (4.5%) for more than 5 h. The short-video platforms used are TikTok (616; 56.6%), Kuaishou (360; 33.1%), Huoshan (108; 9.9%), and other platforms (5; 0.5%). In addition, the participants’ mean age was 19.19 years (standard deviation of 1.07 years).

### 3.3. Measurement

The questionnaire was translated and modified from reliable instruments used in previous studies. The study population of this study comprised students. The questionnaire was evaluated by three educational experts and was measured with a 5-point Likert scale (1 to 5 for strongly disagree to strongly agree).

#### 3.3.1. Short-Video Problematic Use

In this study, the short-video scale from Ye et al. was used as a reference and adapted, with 10 questions [24]. When the average score is higher, it means a higher level of addiction. Example questions are: “I will put aside what I need to finish or do and spend my time watching short videos” and “I will sacrifice my sleep at night by watching short videos”.

#### 3.3.2. Learning Engagement

The study adapted the Learning Engagement Scale of Luan et al., with eight items each to analyze participants’ views of engagement in learning. When the average score is higher, it means a higher level of engagement [35]. An example of behavioral engagement is: “I usually arrive on time for class”; an example of emotional engagement is: “If the quality of my work is not satisfactory after I finish it, I am willing to correct it again”; and an example of cognitive engagement is: “I will remind myself to check the areas of my work where I tend to make mistakes”.

#### 3.3.3. Perceived Learning Ineffectiveness

This study was based on and adapted from Hong et al.’s Learning Ineffectiveness Scale, which consists of eight questions to measure participants’ perceptions of the adverse effects on learning after using short-video applications [18]. The higher the average score, the more severe the ineffectiveness of learning. Example questions are: “Since I started watching short videos, I think my study efficiency has decreased” and “Since I started watching short videos, I think the quality of my homework has deteriorated”.

### 3.4. Data Analysis

Structural equation modeling (SEM) is a statistical modeling tool that has been popularly used in many disciplines [40]. SEM is a theory-driven method for analyzing data and can be conducted to evaluate hypotheses and the causal association between measured and/or possible variables [41]. At the same time, SEM has many advantages, including the fact that when analyzing the association among factors, these associations have no measurement errors because the errors have been estimated or eliminated, leaving only the common variance; and by estimating and eliminating the measurement error, the reliability of the measurements can be stated explicitly in the analysis [42]. Therefore, this study used SEM to validate the research model based on the theoretical framework constructs.

## 4. Results and Discussion

This was a validation study; therefore, the reliability, validity analysis, and descriptive statistics analysis were tested with SPSS 23.0, followed by AMOS 23.0 for item analysis, overall fit analysis, and model validation. The related statistical results are described as follows.

### 4.1. Item Analysis

In order to ensure whether the measurement model has a good fit, we first conducted an item analysis, and the criteria for the relevant fit indicators were that the χ^2^/*df* value must be smaller than the value 5, the RMSEA must be smaller than 0.10, and the GFI and AGFI must be greater than the value 0.80. Questions with a factor loading (FL) not greater than the value 0.50 must be eliminated from the questionnaire [43,44]. Table 1 shows the results of the analysis. Based on the above analysis criteria, items were deleted as follows: short-video problematic use was reduced from ten to seven questions, behavioral engagement from eight to five questions, emotional engagement from eight to six questions, cognitive engagement from eight to six questions, and perceived learning ineffectiveness from eight to six questions.

External validity of the items was used to discriminate the explanatory range of the study [45], and the values of all respondents for each question were divided into the first 27% and the last 27% and *t* tested. If the *t*-value was greater than 3 (*** *p* < 0.001), the external validity was regarded as significant. Table 1 indicates that the *t*-values for the constructs were from 17.90 to 58.57 (*** *p* < 0.001), which means that all of the questions in this study were discriminatory and were able to determine the response level of the different sample groups [46].

### 4.2. Reliability and Validity Analysis

In order to obtain a model that could be used for validation, statistical tests of reliability were performed. Hair et al. suggested that Cronbach’s α composite reliability (CR) values should exceed the criterion of 0.70 [43]. The Cronbach’s α values of the present study components ranged from 0.877 to 0.97, and the CR values ranged from 0.89 to 0.97; thus, both analyses met the suggested criteria, and the results of the analyses are shown in Table 2.

Moreover, Hair et al. also recommended that the FL value must be greater than the threshold 0.50, and if it is smaller than this threshold, the question should be removed [43]. In this study, Table 2 shows the FL values which were between 0.73 and 0.93. Hair et al. indicated that the average variance extracted (AVE) value must be greater than 0.50 to represent the constructs with convergent validity [47]. In this study, the AVE values of the constructs ranged from 0.54 to 0.86. The results of these two analyses also met the suggested criteria, as can be seen in Table 2.

Awang stated that when the threshold of the AVE root number for each construct is greater than the threshold of Pearson’s correlation coefficient for other constructs, this signified that there was discriminant validity for the constructs [48]. The analysis results in this study showed that there was discriminant validity for each construct; that is, all constructs are statistically different and can be used to test the structural model, as shown in Table 3.

### 4.3. Model Fit Analysis

Before performing model validation, it is necessary to verify that the structural model has a suitable degree of compatibility. The relevant fitness metrics are as follows: χ^2^/*df* values must be smaller than the value 5 [43]; RMSEA values must be smaller than 0.1; GFI, AGFI, NFI, NNFI, CFI, IFI, and RFI values must be greater than 0.80 [49]; and PNFI and PGFI values must be greater than 0.50 [43]. This study’s fit index values were χ^2^ = 1670.35, *df* = 400, χ^2^/*df* = 4.18, RMSEA = 0.05, GFI = 0.90, AGFI = 0.88, NFI = 0.95, NNFI = 0.96, CFI = 0.96, IFI = 0.96, RFI = 0.94, PNFI = 0.87, and PGFI = 0.77.

### 4.4. Path Analysis

**Figure 2:**
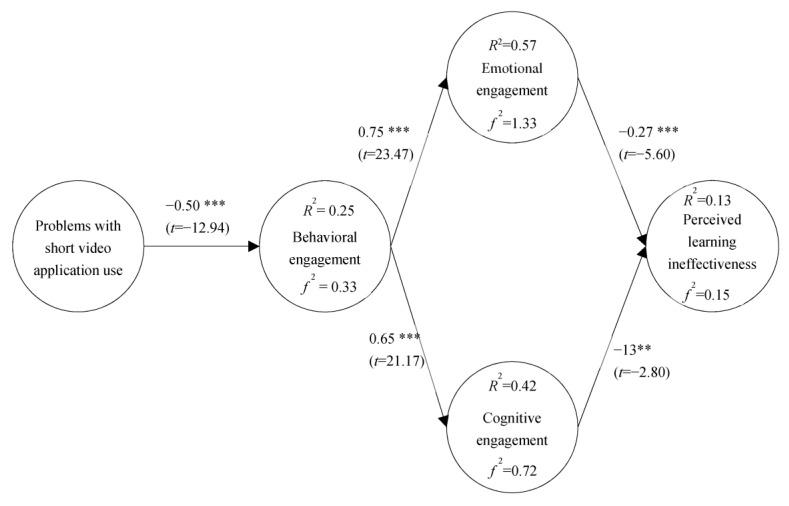
Research model verification. ** *p* < 0.01. *** *p* < 0.001.

The study model validated that short-video problematic use was negatively related to behavioral engagement (β = −0.50 ***; *t* = −12.94); behavioral engagement was positively related to emotional engagement (β = 0.75 ***; *t* = 23.47); behavioral engagement was positively related to cognitive engagement (β = 0.65 ***; *t* = 21.17); emotional engagement was negatively related to perceived learning ineffectiveness (β = −0.27 ***; *t* = −5.60); and cognitive engagement was negatively related to perceived learning ineffectiveness (β = −0.13 **; *t* = −2.80), as can be seen in Figure 2.

In addition, the explanatory power of short-video problematic use on behavioral engagement was 25% with an *f2* of 0.33; the explanatory power of behavioral engagement on emotional engagement was 57% with an *f2* of 1.33; the explanatory power of behavioral engagement over cognitive engagement was 42% and *f2* was 0.72; the explanatory power of emotional and cognitive engagement over perceived learning ineffectiveness was 13%; and *f2* was 0.15, as can be seen in Figure 2.

### 4.5. Discussion

In this study, short-video problematic use was regarded as the inability of participants to use short-video applications in a reasonable manner; it includes the inability to control the use of time, such as still wanting to watch short videos when busy, leading to interference with their daily routine. Behavioral engagement refers to participants’ ability to complete learning tasks in an accurate and responsible manner. Emotional engagement refers to the participant’s sense of identification with and emotional response to the learning task throughout the learning process. Cognitive engagement is defined as the application of a postulated cognitive strategy by the participant during learning. Perceived learning ineffectiveness was defined as the participant’s perception of the learning situation becoming poor after using the short video.

Based on the definitions of the above dimensions and in the cross-sectional analysis, the results of this study showed that the mean of students’ short-video problematic use is 2.34, which is lower than the average (3.00). This indicates that student respondents are less likely to have short-video problematic use; the mean of students’ behavioral engagement is 3.53, the mean of students’ emotional engagement is 3.33, and the mean of students’ cognitive engagement is 3.46, which is higher than the average (3.00). In terms of the mean of the three types of learning engagement, students generally perceived themselves as being better engaged in learning activities. The mean of students’ perceived learning ineffectiveness was 2.83, which was lower than the mean (3.00), indicating that the students generally perceived less learning ineffectiveness occurring.

#### 4.5.1. Short-Video Problematic Use Has a Negative Effect on Behavioral Engagement

The results of this study indicated that short-video problematic use was negatively related to behavioral engagement. That is, the more severe a student’s short-video problematic use is, the less engaged they will be in their learning. Such results can also be well explained by past studies.

According to Perales et al., the process of addiction is a transition between behavioral control patterns [25]. If people are addicted to watching short videos, it will cause them to neglect their normal school life and work, which will have a negative impact on their schooling [3]. Sunday et al. also found that smartphone addiction negatively affects students’ learning [26], and Roberts et al. also indicated that students’ attention in the classroom is disturbed by their phone use, and this distraction further affects their learning outside of the classroom [27]. That is, problematic use will have a negative effect on learning or academics. In addition, Tas’s study found that Internet addiction was a significant predictor of school engagement [28]. From this study, it is clear that short-video problematic use, like other Internet addictions, has the same negative impact on learning and, more specifically, on students’ behavioral engagement.

#### 4.5.2. Behavioral Engagement Has a Positive Effect on Emotional and Cognitive Engagement

In the past, most studies analyzed multidimensional engagements at the same level, rather than examining the causal relationships before and after. In contrast, this study concluded that behavioral engagement should precede emotional and cognitive engagement. The results also confirm that behavioral engagement has a positive effect on affective and cognitive engagement. That is, the better the students’ behavioral engagement in learning, the higher their emotional and cognitive engagement will be.

Such a result can be explained by the arguments and findings of the following scholars. Furthermore, Philp and Duchesne also argued that in different contexts, it is important to emphasize the different dimensions of engagements for the particular outcome sought [31]. Philp and Duchesne noted that when students feel bored or uninterested in a task, they will be emotionally detached from it, and when they are unwilling to listen to other partners’ reactions or engage in interactions, they are less likely to exert effort or persist in learning or to direct their attentional resources in an effective way for cognitive engagement [31]. As seen above, without engagement, students emotionally and cognitively retreat from the learning process [34]. This was confirmed by the study of Luan et al. (2020), who found a role for behavioral engagement in influencing the remaining engagement dimensions [35].

In addition, previous studies have indicated that a strong relationship between behavioral, emotional, and cognitive engagements is often observed. Furthermore, Ben-Eliyahu et al.’s study found that in educational settings, engagement is defined as the process of students’ effective participation in learning activities, and engagement in educational settings is conceptualized as three components: affective, behavioral, and cognitive, which are conceptually separate but interrelated [32]. In summary, when students have good behavioral engagement in learning, it will lead to good emotional and cognitive engagement.

#### 4.5.3. Emotional and Cognitive Engagement Have a Negative Effect on Perceived Learning Ineffectiveness

This study showed that emotional and cognitive engagement were negatively related to perceived learning ineffectiveness. In other words, the more emotionally and cognitively engaged students are in their learning, the less likely they are to be ineffective after using short videos.

This result can also be explained by the following research findings. Pickering noted that student engagement is considered to have a clear and potential relationship with assessment results [36]. Tulaskar and Turunen also indicated that student engagement has a crucial influence on maintaining students’ association with their courses and learning [13]. That is, students’ academic engagement may be an important personal factor that is closely related to academic achievement [29]. Moreover, Hong et al. suggested that once students are well engaged, student learning ineffectiveness is somewhat mitigated [37].

Furthermore, Dolan argued that people are more likely to prioritize events that they perceive as valuable and that the way to achieve this valuation goal is to enhance attention through emotion. Therefore, when people are emotionally engaged, they will give more attention to tasks other than learning and are more likely to achieve good learning outcomes [38]. In addition, Pickering stated that a cognitively engaged student would be one who is willing to engage in learning and take on the challenge of acquiring new knowledge or skills and exerting effort beyond the requirements of the course [36]. Moreover, according to Dubovi and Tabak, students are mentally engaged when they put cognitive effort into understanding, exceeding the demands of the activity, solving problems flexibly, and selecting challenging tasks [39]. Therefore, students’ self-reflection (postulated cognitive ability) can enhance their learning strengths and improve their learning outcomes [16]. According to this, it can be seen that the higher the level of students’ cognitive engagement, the better their learning tasks will be, and the better their learning outcomes will be.

#### 4.5.4. Implications

The convenience of entertainment and the highly immersive feeling that short videos bring to people make the issue of short-video usage a new challenge to overcome. From these results, short-video problematic use shows a negative impact on learners’ behavioral engagement. However, students are often unaware of the seriousness of this outcome for their learning. Although students are legally adults, parents and teachers should still actively understand their usage habits and explain to them the principles of reasonable use to avoid problematic usage behaviors. In addition, according to Adan, establishing a regular day/night pattern is also an effective way to prevent addictive behaviors [50]. Therefore, it is recommended to advocate the daily work and rest of student users to avoid the problem of work and rest disorders due to the use of short-video software.

In addition, because of the adverse effects of addiction on students, platform operators have an obligation to prevent students from using the existing problems and should design some prevention and control mechanisms to fulfill their corporate social responsibility. At the same time, in order to prevent students from watching short videos as their main leisure activity, schools should organize more leisure activities and student clubs that are beneficial to physical and mental health, so that students can focus away from these addictions and build good interpersonal relationships with their peers.

In addition, the results of the study indicated that learning engagement was positively related to learning and can effectively inhibit the occurrence of ineffective learning. Therefore, teachers should adopt a learner-centered teaching approach and make good use of innovative teaching strategies to create a meaningful learning environment for students in order to promote active participation in classroom activities and further enhance the three types of learning engagement.

#### 4.5.5. Research Limitations and Future Study

Some limitations were present in this study. First, this study used a cross-sectional design to conduct a questionnaire survey, and these data were used for statistical analysis. Therefore, the results obtained show the relationship of the variables and only capture the perceptions of the participants at this point in time but not the longitudinal association between short-video problematic use and other learning factors. Therefore, it is suggested that future research could adopt a longitudinal design to track short-video-addicted users over a long period of time, so as to confirm that poor learning engagement and ineffective learning are both the result of problematic use of short videos. In addition, the data collection was based on the participants’ personal preferences, without many conditions being set for participation, which may have led to uneven sample characteristics. This is also an issue that can be considered in follow-up studies. In addition, this study was a form of backward extrapolation to determine whether short-video problematic use would cause adverse learning effects. Therefore, it is possible that this is a symptom of social media/short-video addiction rather than the entire cause of poor learning. Therefore, subsequent studies may expand the exploration of the causes of poor learning.

Secondly, the sample of students in this study was from vocational colleges in China, although the outcomes of the external validity analysis indicated that the results can be extrapolated to different scopes and contexts. However, it is still not possible to compare whether there are differences in the perception of these constructs across school age groups, systems, and regions of the country. Therefore, it is suggested that future studies could extend the sampling area of the study and conduct a difference analysis.

Finally, the self-reporting of participants may have resulted in biased results in order to meet social expectations, leading to a distortion of the descriptive statistics. Moreover, biased responses may be found in this study because it was a relations study that aimed to examine the relationship between pathways, not a descriptive statistical survey [9]; therefore, the impact is limited. However, given the discrepancy between the options selected by the users in the background data and the responses given to the short-video problematic use scale, interviews can be used in the follow-up study to investigate whether this phenomenon is due to the response of the short-video users or the gap in their personal perceptions.

## 5. Conclusions

Recently, the reputation of short videos has been spreading rapidly. Whether it is active use (creators) or passive use (viewers), the problem of short-video problematic use has gradually become an Internet addiction-related issue of concern to the general public, educators, and even researchers. However, compared to other Internet addictions, short-video problematic use is an emerging area of research, and there are still few empirical studies on the effects of short-video problematic use on young students. This is an essential concern that needs to be extensively explored. Therefore, for this study, we constructed a research model within the framework of EST and engagement theory to extend the understanding of the influence of short-video problematic use in the education field; this is a significant contribution of this study to the Internet addiction field. The results in this study indicated that: 1. short-video problematic use was negatively related to behavioral engagement; 2. behavioral engagement was negatively related to both emotional and cognitive engagement; and 3. both emotional and cognitive engagement have a negative effect on perceived learning ineffectiveness. As shown above, short-video problematic use is also a harmful online behavioral addiction for learning, so it should be avoided or improved by all means. From the study results, it can be seen that behavioral engagement is an important prerequisite for promoting emotional and cognitive engagement, and short-video problematic use is a key antecedent of behavioral engagement.

## Figures and Tables

**Table 1:** Confirmatory factor analysis.

Index	χ^2^	*df*	χ^2^/*df*	RMSEA	GFI	AGFI	FL	*t*
Threshold	---	---	<5	<0.10	>0.80	>0.80	>0.50	>3
Short-video problematic use	58.69	14	4.19	0.05	0.98	0.97	0.56~0.86	18.69~ 40.31
Behavioral engagement	20.53	5	4.11	0.05	0.99	0.98	0.57~ 0.92	17.90~ 58.57
Emotional engagement	37.95	9	4.22	0.05	0.99	0.97	0.79~ 0.91	30.73~ 38.20
Cognitive engagement	36.19	9	4.02	0.05	0.99	0.97	0.78~ 0.90	32.68~ 42.64
Perceived learning ineffectiveness	43.27	9	4.81	0.06	0.99	0.97	0.90~ 0.94	38.97~ 47.00

**Table 2:** Reliability and validity analysis.

Constructs	*M*	*SD*	α	CR	AVE	FL
	---	---	>0.70	>0.70	>0.50	>0.50
Short-video problematic use	2.34	0.91	0.87	0.89	0.54	0.73
Behavioral engagement	3.53	0.92	0.91	0.91	0.68	0.82
Emotional engagement	3.33	0.83	0.94	0.94	0.74	0.86
Cognitive engagement	3.46	0.82	0.94	0.94	0.73	0.85
Perceived learning ineffectiveness	2.83	0.97	0.97	0.97	0.86	0.93

**Table 3:** Discriminant construct validity.

Construct	1	2	3	4	5
1. Short-video problematic use	(0.85)				
2. Behavioral engagement	−0.45	(0.91)			
3. Emotional engagement	−0.25	0.73	(0.93)		
4. Cognitive engagement	−0.19	0.60	0.77	(0.92)	
5. Perceived learning ineffectiveness	0.27	−0.35	−0.35	−0.32	(0.96)

Note: The value on the diagonal line is the square root value of AVE, whereas the other values are the related coefficient values.

## Data Availability

The original contributions presented in the study are included in the article.
